# Geriatric Surgery Produces a Hypoactive Molecular Phenotype in the Monocyte Immune Gene Transcriptome

**DOI:** 10.3390/jcm12196271

**Published:** 2023-09-28

**Authors:** Rachel L. Oren, Rachel H. Grasfield, Matthew B. Friese, Lori B. Chibnik, John H. Chi, Michael W. Groff, James D. Kang, Zhongcong Xie, Deborah J. Culley, Gregory Crosby

**Affiliations:** 1 Cognitive Outcomes of Geriatric Surgery Research Center, Department of Anesthesiology, Perioperative and Pain Medicine, Brigham and Women’s Hospital, Boston, MA 02115, USA; rachel.oren@yale.edu (R.L.O.); rachel.grasfield@gmail.com (R.H.G.); 2 Translational Medicine and Clinical Pharmacology, Sanofi, Cambridge, MA 02139, USA; mfriese@gmail.com; 3 Department of Epidemiology, Harvard T.H. Chan School of Public Health, Boston, MA 02115, USA; 4 Department of Neurosurgery, Brigham & Women’s Hospital, Harvard Medical School, Boston, MA 02115, USA; jchi@bwh.harvard.edu (J.H.C.); mgroff@bwh.harvard.edu (M.W.G.); 5 Department of Orthopedic Surgery, Brigham & Women’s Hospital, Harvard Medical School, Boston, MA 02115, USA; jdkang@bwh.harvard.edu; 6 Geriatric Anesthesia Research Unit, Department of Anesthesia, Critical Care and Pain Medicine, Massachusetts General Hospital, Harvard Medical School, Charlestown, MA 02129, USA; zxie@mgh.harvard.edu; 7 Department of Anesthesiology and Critical Care, University of Pennsylvania Perelman School of Medicine, Philadelphia, PA 19104, USA; deborah.culley@pennmedicine.upenn.edu; 8 Cognitive Outcomes of Geriatric Surgery Research Center, Department of Anesthesiology, Perioperative and Pain Medicine, Brigham & Women’s Hospital, Harvard Medical School, Boston, MA 02115, USA

**Keywords:** aging, surgery, inflammation, gene expression, monocytes

## Abstract

Surgery is a major challenge for the immune system, but little is known about the immune response of geriatric patients to surgery. We therefore investigated the impact of surgery on the molecular signature of circulating CD14^+^ monocytes, cells implicated in clinical recovery from surgery, in older patients. We enrolled older patients having elective joint replacement (N = 19) or spine (N = 16) surgery and investigated pre- to postoperative expression changes in 784 immune-related genes in monocytes. Joint replacement altered the expression of 489 genes (adjusted *p* < 0.05), of which 38 had a |logFC| > 1. Spine surgery changed the expression of 209 genes (adjusted *p* < 0.05), of which 27 had a |logFC| > 1. In both, the majority of genes with a |logFC| > 1 change were downregulated. In the combined group (N = 35), 471 transcripts were differentially expressed (adjusted *p* < 0.05) after surgery; 29 had a |logFC| > 1 and 72% of these were downregulated. Notably, 21 transcripts were common across procedures. Thus, elective surgery in older patients produces myriad changes in the immune gene transcriptome of monocytes, with many suggesting development of an immunocompromised/hypoactive phenotype. Because monocytes are strongly implicated in the quality of surgical recovery, this signature provides insight into the cellular and molecular mechanisms of the immune response to surgery and warrants further study as a potential biomarker for predicting poor outcomes in older surgical patients.

## 1. Introduction

Surgical trauma produces a profound peripheral immune response that is essential for recovery but is also implicated in the development of complications and suboptimal outcomes. This intricate programmed response is activated within hours of tissue injury and engages multiple types of immune cells, which are recruited to the site by damage-response antigens, alarmins, and increased levels of pro-inflammatory cytokines [1,2,3,4,5,6]. The transcriptome of the cells also changes widely; after severe blunt trauma, for example, over 80% of the leucocyte transcriptome is altered within 28 days of the injury [7]. Moreover, gene expression analyses demonstrate that pathways associated with the adaptive immune system are downregulated as a result of surgery while, conversely, pathways associated with the innate immune systems are upregulated [8]. In some cases, these increasingly detailed investigations of the immune response to trauma and surgery have identified molecular correlates of clinical recovery [8,9]. Thus, though mechanistic relationships remain to be established [4,6,8,9,10], the immune state appears to personalize risk [1].

Nowhere is this issue more relevant than in geriatric surgery. Older patients are a demographic that is under-represented in studies of trauma or surgery, including those examining the immune response. Despite representing just 15% of the population, older adults constitute over 50% of all surgical admissions and, on a per capita basis, are nearly three times more likely to have surgery than a middle-aged individual [11]. Likewise, older adults have more comorbidities, are at greater risk of having surgical complications and poor outcomes than younger adults [12,13], and their outcomes are worse [11,14,15,16]. For example, even after elective procedures such as joint replacement or spine surgery that are intended to mitigate pain and restore function, a sizeable subset of older patients experience serious morbidity including delirium and slow return to independence [17,18]. The reasons for this are not well understood, but age-related differences in the immune response to surgery likely play a role, as both initiation and resolution of inflammation become dysfunctional with age [19,20,21,22]. Thus, proinflammatory cascades tend to be active, resulting in elevated plasma cytokines such as IL6 and MCP-1, and, even in the absence of infection or stress, macrophage function is compromised, and the transcription and function of myeloid cell subsets changes [19,23]. The capacity to resolve inflammation is probably also deficient in aging as basal levels of pro-resolving lipid mediators (e.g., resolvin D1) and the ability to upregulate resolution programs in response to an immune challenge appear to decline [20,24,25,26,27]. This is important in a surgical setting because resolution mediators resolve inflammation naturally without inhibiting inflammation or producing immune suppression [24,28]. Together, this imbalance between immune activation and immune paralysis leaves older patients immunocompromised and more susceptible to infection and complications after surgery [29,30,31,32].

Cellular immunity is a critical component of the response to injury. There are significant changes in circulating leukocyte populations within hours of a surgical incision, and the changes last for at least a day [33]. Monocytes, which tend to increase in number after surgery, are particularly relevant because they are major sensors, coordinators, and mediators of immunity [34,35]. Previous work demonstrates a strong correlation between signaling responses in these cells and the quality of clinical recovery and development of serious surgical morbidity [9,16,36]. For example, the activation state of monocytes correlates with fatigue, pain, and functional impairment of the operated joint after hip surgery and explains as much as 60% of the between-patient variability in the rate of recovery [9]. Furthermore, monocytes are implicated in the development of postoperative cognitive morbidity such as delirium, which is one of the most common postoperative complications in older surgical patients [37,38,39,40,41]. Yet, most research has focused on plasma markers of inflammation rather than the response of specific circulating immune cells. Nor have previous studies comprehensively examined the impact of surgery on the transcriptome of cell types strongly incriminated in adverse surgical outcomes in older patients. Accordingly, we conducted an exploratory study to examine and define the impact of geriatric surgery on the molecular signature of circulating CD14^+^ monocytes. We hypothesized that surgery induces numerous changes in the immune gene transcriptome of these cells, with the profile having features of both immune activation and suppression, and that some aspects of the signal are conserved across different surgical procedures. To test these possibilities, we examined the expression of 784 inflammation-related genes in the circulating monocytes of older patients before and after elective spinal or total hip or knee replacement surgery, procedures that are among the most common in this age group. Our results reveal a replicable effect of elective geriatric surgery on expression of immune-related genes in monocytes, with many changes consistent with development of a hypoactive/immunocompromised phenotype postoperatively.

## 2. Methods

### 2.1. Study Participants

The Partners Institutional Review Board (IRB) approved two prospective observational studies (2016P000012 and 2014P002400) from which these data were obtained. Study participants were aged 65 years and older and scheduled for elective surgery at Brigham and Women’s Hospital (Boston, MA, USA) with planned hospital admission of at least one night. One group consisted of patients scheduled for lower extremity total joint replacement surgery (hip or knee) and were recruited between 1 February 2016 and 30 March 2017. Another group included patients scheduled for elective spinal surgery (cervical, thoracic, lumbar, or sacral/pelvic), and were recruited between 22 February 2016 and 9 October 2018. Details have been reported previously [17,18]. We selected these procedures because they are among the most common elective procedures performed in this age group in the US [10]. For both cohorts, all eligible patients were identified through the EPIC EMR system the day before their preoperative appointment. Patients who met at least one of the following criteria were excluded from the current study: postoperative planned ICU admission, history of stroke or brain tumor, uncorrected vision or hearing impairment (specifically, inability to see pictures or read or hear instructions), limited use of the dominant hand (specifically, limited ability to draw), or inability to speak, read, or understand English. After a research team study member obtained written informed consent, demographic and perioperative characteristics were collected from medical records: age; weight; BMI; sex; highest level of education; and type of surgical procedure, estimated blood loss, hospital length of stay, and 6-month mortality. This exploratory study included 35 randomly selected patients (19 joint replacement, 16 spine surgery) that had both preoperative and postoperative day 1 blood samples sufficient for isolation of CD14^+^ cells.

### 2.2. Sample Collection

Preoperative blood samples were collected along with routine laboratory studies drawn during the patients’ preoperative appointment (preop). If routine laboratory studies were not collected during the preoperative appointment, a preoperative sample was collected prior to entry into the operating room on the day of surgery. The postoperative blood sample was collected by study staff on postoperative day 1 (postop). Postoperative day 1 was chosen as the postoperative time point because several other studies have found the greatest appreciable differences in inflammatory markers at this time [15,19,20]. Samples were collected using a 21-gauge needle vacutainer (BD Vacutainer, Becton Dickinson, Franklin Lakes, NJ, USA) into 10 mL ethylenediamine tetra-acetic acid (EDTA) anticoagulant tubes and were processed within 30 min.

### 2.3. Isolation of CD14^+^ Monocytes

CD14^+^ monocytes were isolated using the positive immunomagnetic selection kit from Miltenyi Biotec according to the manufacturer’s instructions. A total of 2 mL of whole blood and 100 µL of StraightFrom Whole Blood CD14^+^ MicroBeads (Miltenyi Biotec, Bergisch Gladbach, Germany) were incubated at 4 °C for 15 min. Then 3 mL of phosphate-buffered saline (PBS) containing 0.5% bovine serum albumin (BSA), and 0.4% EDTA was added and the mixture was spun at 450 g for 10 min. Plasma was aspirated, and another 1 mL of buffer was added. The cell suspension was loaded onto an LS magnetic column (Miltenyi Biotec, Bergisch Gladbach, Germany) placed in the magnetic field of a MACS Separator (MIDIMACS) and rinsed three times with buffer. The column was then removed from the magnetic field, and the CD14^+^ fraction was eluted in a total volume of 5 mL. Cell concentrations were taken to ensure accurate and sufficient cell count using a Countess II FL Automated Cell Counter (Life Technologies, Carlsbad, CA, USA). Cell counts in the range of 1.0 × 10^5^ were considered accurate and acceptable. The fraction was then centrifuged at 450× *g* for 10 min, elution buffer was aspirated, and the cells were lysed with 600 µL of Buffer RLT (Qiagen, Hilden, Germany; catalog #79216). Lysate was then frozen at −80 °C until RNA was isolated.

### 2.4. RNA isolation and Quality Assessment

Total RNA from CD14^+^ cells was isolated and purified using the Qiagen RNeasy mini kit (Hilden, Germany; Catalog #74106) according to the manufacturer’s instructions. RNA was isolated form all samples for each cohort at once to limit potential batch effects. Following RNA isolation, total RNA was quantified with an A260/A280 ratio via a Nanodrop ND-1000 spectrophotometer (NanoDrop, Wilmington, DE, USA) for a few samples at random to confirm extraction efficacy and expected concentration. Isolated RNA samples were then sent for a more robust quality assessment with an Agilent 2100 Bioanalyzer (eukaryote total RNA Nano, version 2.6, Agilent Technologies, Santa Clara, CA, USA) for determination of total eukaryotic RNA concentration and the RNA integrity number (RIN). All samples had a spectral 260/280 ratio of between 2.05 and 2.13 and an RIN of at least 8, with most samples above 9.5.

### 2.5. NanoString

Samples were diluted to 20 ng/µL based on dilutions provided by the Bioanalyzer. Isolated RNA samples were sent to the NanoString nCounter^®^ platform at the Center for Advanced Molecular Diagnostics, a Brigham and Women’s Hospital Core Facility. The “PanCancer Immune Profiling Panel” panel of 770 genes was chosen, and 14 unique genes were added for a total microarray of 784 immune-related genes. Probe sets for each gene were designed and synthesized by NanoString nCounter^®^. Technical replicates of samples were used to ensure consistency within and between batches. There were no significant differences between or within batches.

### 2.6. Statistical Analysis

All analyses were performed in RStudio (Version 1.2.1335 for MacOS) using the R programming language. We used *t*-tests or Wilcoxon-tests for continuous variables where appropriate and Chi-square tests for categorical variables to examine demographic and clinical data. We utilized the Linear Models for Microarray Data “limma” R package to screen for pairwise comparisons of gene expression between preoperative and postoperative samples [42]. The voom function in limma was used to normalize count data into counts per million (CPM) [43], and we formulated the design to cluster on subject in order to account for repeated measures. We adjusted *p*-values for false discovery rate (FDR) using Benjamini–Hochberg (BH) adjustment, with the cutoff set at 0.05. Genes that met the adjusted *p*-value cutoff were defined as differentially expressed genes (DEGs). We also applied an absolute log2 fold change (logFC) > 1 as an additional cut-off criterion of biological relevance. Visualizations were configured in “Gplots”, “EnhancedVolcano”, and/or “pheatmap” R language packages.

## 3. Results

The joint replacement (N = 19) and spine surgery (N = 16) groups were comparable in age (75.6 ± 5.4 vs. 74.6 ± 4.5 years, respectively; *p* > 0.5), BMI, and education, and in clinical and outcome variables including estimated blood loss and length of stay (Supplementary Tables S1 and S2). Most joint replacement patients were female, but the opposite was true for those having spine surgery (63% and 37.5% female, respectively; Supplementary Table S1). All subjects in the spinal surgery group had general anesthesia, whereas only 63% of the joint replacement procedures involved a general anesthetic (Supplementary Table S2). Subjects in the joint replacement group were nearly equally divided between knee (N = 10) and hip arthroplasty (N = 9). Likewise, the spinal surgeries were equally divided between tier 2 (N = 8; less invasive (e.g., laminectomy, facetectomy)) and tier 3 (N = 8; more invasive (fusion, trauma) procedures).

**Figure 1:**
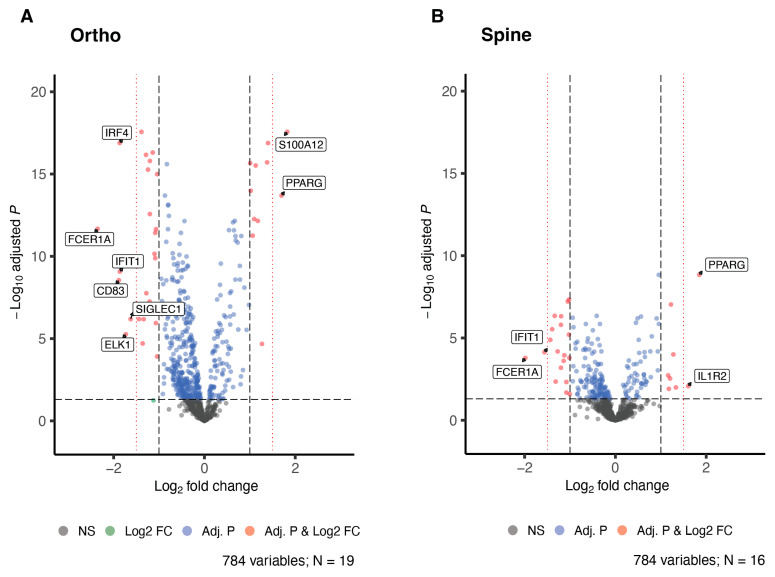
Volcano plots of differential mRNA expressions as a result of (**A**) hip or knee replacement surgery and (**B**) spinal surgery. The x-axis is the log2 fold-change, and the y-axis is the negative logarithm of the BH-adjusted *p*-values. Red dots denote differentially expressed genes (DEGs) with a BH-adjusted *p* < 0.05 and a |logFC| > 1.0; blue dots denote DEGs by H-adjusted *p*-value but not the |logFC| > 1 threshold. Additional threshold lines (red, dotted) at ±1.5 logFC are depicted to highlight the DEGs with a log2FC of greater than 1.5.

We examined pre- to postoperative changes in expression of 784 transcripts relevant to the immune response in CD14+ monocytes. Joint replacement surgery produced preoperative to postoperative changes in the expression of 489 genes (62% of 784; BH-corrected *p* < 0.05). Of these DEGs, 38 (7.8%) had a |logFC| > 1 (i.e., >100% change) in the postoperative period compared to their preoperative baseline expression, and 8 (1.6%) exhibited at least a 1.5 logFC (i.e., a 225% increase or decrease; Figure 1A). Of the 38 transcripts with a |logFC| > 1, 11 (29%) were upregulated and 27 (71%) were downregulated.

In the spine surgery patients, 209 (27% of 784) of the genes on the panel were differentially expressed after surgery (BH-corrected *p* < 0.05). Of these DEGs, 27 (12.9%) had a |logFC| > 1 postoperatively compared to the preoperative baseline expression, and 4 had at least a 1.5 logFC (Figure 1B). As in the joint replacement group, of the transcripts with a |logFC| > 1, the majority (19 [70%]) were downregulated postoperatively.

**Figure 2:**
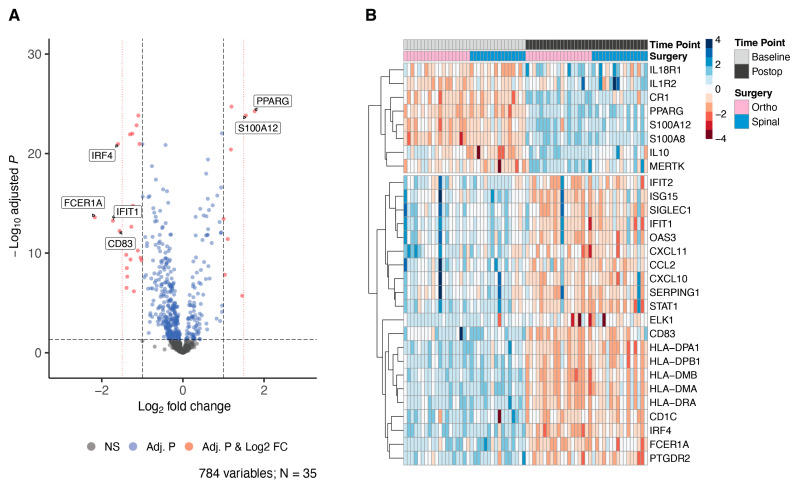
Volcano plot and heatmap of the genes differentially expressed in the combined cohort (hip or knee replacement, spinal surgery) after surgery. (**A**) Volcano plot of mRNA expression. The x-axis is log2 fold-change, and the y-axis is the negative logarithm of the BH-adjusted *p*-values. Red dots denote the 29 differentially expressed genes (DEGs) with a BH-adjusted *p* < 0.05 and a |logFC| > 1.0; blue dots denote DEGs by BH-adjusted *p*-value but not the |logFC| > 1 threshold. Additional threshold lines (red, dotted) at ±1.5 logFC show the six DEGs with >2.25-fold change in expression. (**B**) Heatmap of the pre- and postoperative expression of 29 DEGs (BH-adjusted *p* < 0.05, |logFC| > 1.0). Values are z-scores; red indicates below average expression, and blue indicates above average expression.

**Figure 3:**
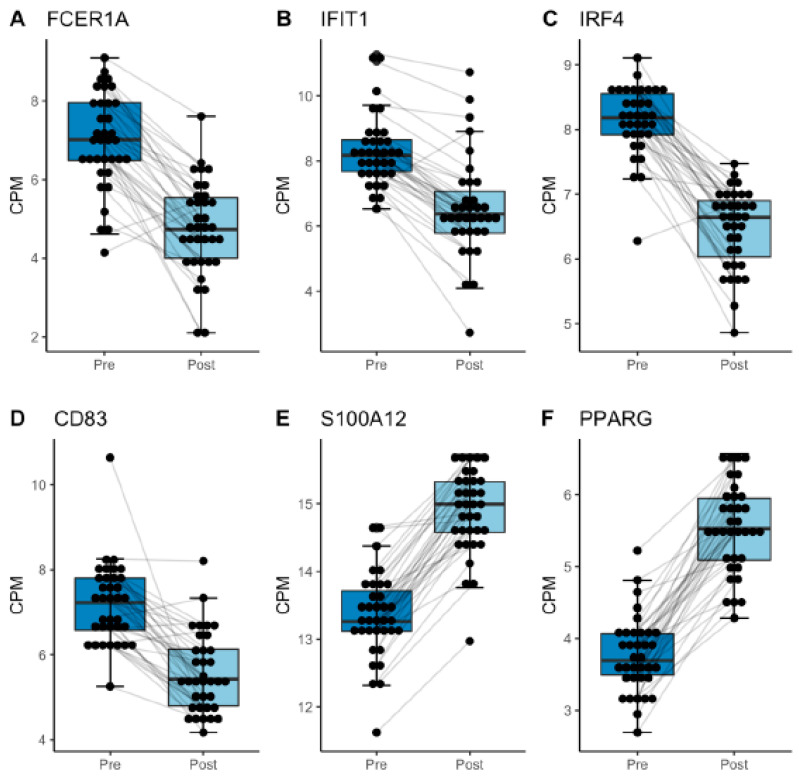
Boxplots of surgery-induced change in expression of selected DEGs (absolute logFC ≥ 1.5; BH-adjusted *p* < 0.05) in the combined cohort. Expression of (**A**) FCER1a, (**B**) IFIT1, (**C**) IRF4, and (**D**) CD83 decreased significantly after surgery, whereas (**E**) S100A12 and (**F**) PPARG increased markedly. Data are normalized preoperative and postoperative counts (logCPM).

**Figure 4:**
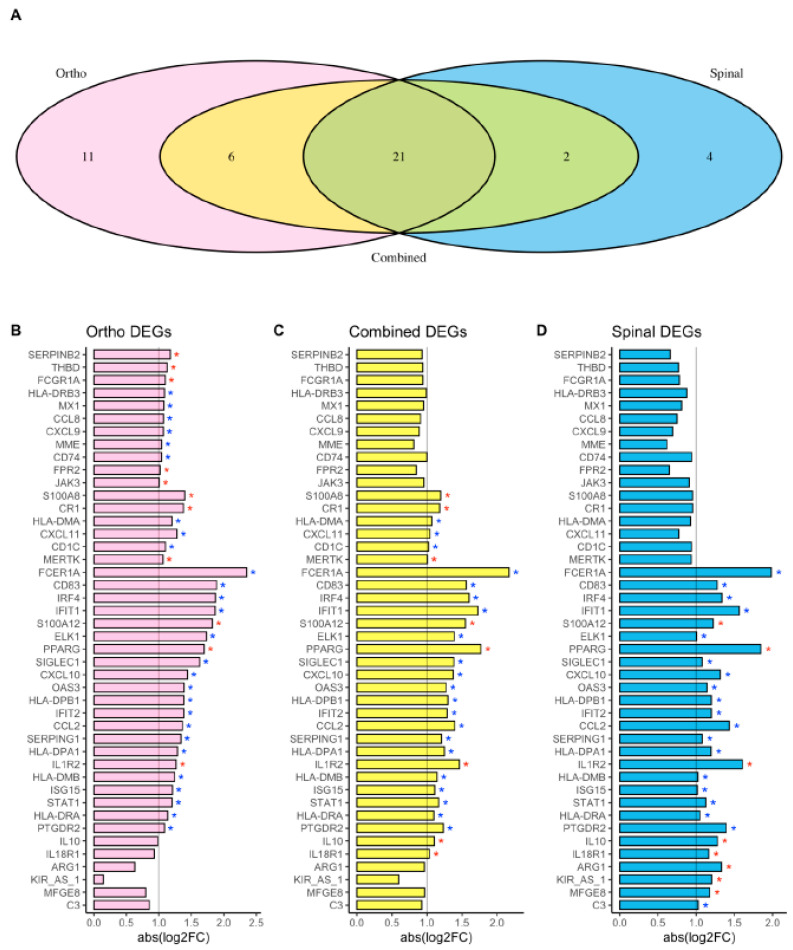
DEGs by surgical procedure type and in the combined group. (**A**) Venn diagram illustrating the number of DEGs in the discovery (hip or knee replacement), replication (spine), and combined cohorts. Each procedure has unique DEGs, but 21 are common to both (dark green oval). (**B**–**D**) Bar plots depicting significantly differentially expressed genes in the (**B**) joint replacement (pink), (**C**) combined (yellow), and (**D**) spinal surgery (blue) groups. The *x*-axis is the absolute value of the log2FC for each gene; no gene had directionally opposite values (e.g., upregulation in one cohort, downregulation in the other). The vertical line on each bar chart shows threshold for significance (log2FC > 1 or < −1). * indicates BH-adjusted *p*-value < 0.05 for the given analysis; a red * indicates the gene was upregulated and a blue * indicates downregulation.

Given the similarities in demographics and clinical characteristics of the groups, we combined the data sets and analyzed them as a single group. This revealed that 471 genes (60% of 784) were differentially expressed (BH-corrected *p* < 0.05) as a result of elective surgery. Of these DEGs, 29 transcripts (6.2%) had a |logFC| > 1 change (Figure 2A), and more were downregulated than upregulated (21 [72%] vs. 8 [28%], respectively; Figure 2B). Six genes had at least a 1.5 logFC change after surgery (Figure 3); four were downregulated and two were upregulated by surgery. Of note, several genes were unique to the joint replacement (n = 11) or spinal surgery (n = 4) groups, but 21 DEGs were common to the different procedures and the combined group (Figure 4, Table 1).

## 4. Discussion

This exploratory study demonstrates that surgery in the older patient produces widespread changes in the molecular signature of circulating CD14^+^ monocytes. Specifically, on a large panel of genes that covers both the adaptive and innate immune response, 21–49% of the genes in these cells were differentially up- or downregulated 24 h postoperatively. A smaller subset of those were changed by at least 1 logFC (e.g., 6.2% of the DEGs in the combined cohort)—a threshold compatible with biological relevance. Most strikingly, among the DEGs with at least a 1 logFC change, 21 were common to the joint replacement, spine, and combined groups. This commonality suggests these molecular events reflect general aspects of surgery, such as tissue injury and stress, more than the procedural specifics, though a larger data set is necessary to prove this assertion. The fact that the majority of the DEGs with at least a 1 logFC change were downregulated suggests that surgery triggers an imbalance in the immune homeostasis of these cells, likely shifting them toward a hypoactive phenotype. Further work is needed to establish such a functional correlate, but the data presented here support previous studies showing that monocyte dysfunction occurs postoperatively [9,44,45,46].

Numerous investigations of the immune response to surgery or tissue injury reveal that blunt trauma or surgery induce a consistent and robust immune response. This is measurable in plasma and/or circulating immune cells and is both rapidly activated and persistent [7]. However, many of the surgical studies involved a single procedure [2,4,6,9,46,47,48] or grouped procedures with varying degrees of invasiveness [3,8], examined a limited number of transcripts or proteins, or included only young or middle-aged subjects. The latter is especially relevant because the results may not translate to an older surgical population that has multiple age-related changes in the immune system [19,20,21,22]. Ours is one of the few studies to focus on older patients and surgical procedures common to that age group and to examine surgery-induced changes in the transcriptome with multiplexed methods. It also appears to be the only study to do so in a single cell type (monocytes) during different surgical procedures. One other study used a similar approach, though in a different type of surgery and different cells [8]. That work, which involved open esophagectomy or pancreaticoduodenectomy, whole-blood leucocytes, and slightly younger patients (66 ± 11 years), found that about 80% of the nearly 41,000 leucocyte genes analyzed undergo changes in expression with surgery [8]. Of these, 522 (1.2%) demonstrated a twofold change, with half being upregulated and the rest downregulated [8]. There also was no association between gene expression changes and procedural variables (procedure type, surgery duration, blood loss). Despite differences in study design (older patients, joint replacement and spine surgery, monocytes), our results generally replicate those findings in that we found surgery altered the expression of a large percentage of the genes analyzed and that more were downregulated than upregulated. It is also notable that four of the DEGs in our combined cohort that exhibited a >1.5 logFC (FCER1a, S100a12, IFIT1, IL1r2) were also significantly changed in the abdominal surgery patients [8]. This is consistent with studies that show remarkably similar changes in global gene expression between patients and across insults of varying type and injury burden [7], implying that—at least in older patients—injury is injury as far as the immune system is concerned. With that in mind, the small subset of DEGs common to both surgical procedures are worthy of further study as potential biomarkers for predicting the immune response to surgery in older patients and as potential therapeutic targets for modifying it in order to improve outcomes.

Our analysis focused on transcripts that have a role in mediating and fine tuning the immune and inflammatory response, but their role in surgical recovery and morbidity is largely unexplored. Peroxisome proliferator-activated receptor gamma (PPARγ), the most robustly upregulated transcript, increases the capacity of macrophages to engulf apoptotic neutrophils [49] and is an initiating protein in homeostatic pro-resolving cellular responses in some models [50]. S100a12, a calcium binding protein that mediates pro-inflammatory processes via RAGE (receptor for advanced glycation end-products) and complement receptor 1 (CR1), which controls activation of the complement cascade, were similarly upregulated [51,52,53,54]. These results imply that surgery stimulates the proinflammatory function of CD14^+^ cells, but changes in other transcripts suggest anti-inflammatory pathways are also perturbed. IL1R2, which serves as a decoy receptor and molecular trap for the proinflammatory cytokine IL1, increases, as does IL-10, one of the most potent anti-inflammatory cytokines [46]. In addition, some molecules that normally counter-balance the inflammatory response to tissue injury or infection are downregulated. One is CD83, which helps orchestrate the balance between protective immunity and harmful autoimmunity. This is relevant because, in animal models, ablation of CD83 yields a proinflammatory phenotype in dendritic cells and impairs resolution of inflammation [55], a process already deficient in the aged [20]. Likewise, IFIT1, an important regulator of the innate immune response to microbial challenge [48], is downregulated. Overall, these results imply that monocytes develop a hypoactive profile postoperatively. This is consistent with evidence, in patients similar to ours in terms of age and procedure types, that surgery decreases receptors on monocytes that regulate the stimulatory response [33] and reduces production of inflammatory mediators by these cells postoperatively [45]. The clinical significance of the changes we identified remains to be determined, but the fact that monocytes play a critical role in the response to tissue injury and clinical recovery from surgery [9,16] makes the observations relevant for enhancing risk-prediction and, potentially, for improving surgical outcomes in this vulnerable group via biomarker targeted immunotherapeutics.

Our study has notable strengths, including analysis by within-subject change that offsets variation in baseline inflammatory status or genetic factors, use of a large but focused panel of genes, and the high prevalence and relative homogeneity of the surgical procedures within the joint replacement and spinal surgery groups. Nonetheless, it also has important limitations. First, the study is small, and we sampled at only one postoperative time point. This prevented an examination of associations between gene expression changes and surgical outcomes. It also means we cannot exclude the possibility that some biologically and clinically relevant changes in gene expression were missed or that they occurred later in the recovery process. Differences in the sex of the patients as well as the anesthetic agents used could also influence the outcomes, as both have an impact on the immune response. However, the fact that the molecular signature of monocytes was similar in the spine and joint replacement surgery patients, and that four of the 20 DEGs common to both were detected in a previous study of abdominal surgery, suggests the molecular signature we identified is partially conserved across sex, anesthetics, and procedures. Second, we focused on older patients and do not have a younger comparator group. Consequently, although older patients have considerably more postoperative morbidity than younger persons, and much of it is thought to be inflammation-related, we cannot say definitively that the changes are specific to older surgical patients or the specific morbidities of these procedures. However, at least in the case of postoperative delirium, which is one of the most common morbidities of geriatric surgery, the plasma proteomic signature is age-dependent [56]. Third, due to the exploratory nature of the study, we purposefully chose a conservative statistical threshold for biological relevance of >1.0 log2FC, which has been used elsewhere for analyses of NanoString data. The intent was to reduce false positives and eliminate transcripts of less biological relevance, but this cutoff, while likely to capture genes with the greatest biological impact, increases the risk of generating false negatives and missing some potentially important transcripts. Fourth, the study was performed at a single large, urban teaching hospital with patients scheduled for elective surgery, and, thus, may not be generalizable to other hospital settings or procedures. Likewise, because we concentrated on monocytes, our results may not be generalizable to other types of leucocytes. However, we chose monocytes because they are critical for the initial response to and recovery from tissue injury and because their immune signature correlates closely with outcomes of surgery [9,16,46]. Lastly, we utilized a curated microarray over full RNA sequencing because the former has lower stochastic variability and outperforms sequencing in terms of reproducibility and cost [57]. The disadvantage of microarrays, and, particularly, pre-curated arrays like the inflammatory panel used here, is that a limited number of genes often precludes pathway analyses. As such, this work requires validation in a larger sample using more comprehensive molecular approaches, such as RNAseq, that include non-immune transcripts and allow for pathway analyses. Despite these limitations, our results suggest that surgery elicits a relatively consistent signal in the monocyte transcriptome of older patients that includes several novel genes that warrant further investigation.

In summary, in this exploratory study, we identified a broad effect of elective spine and joint replacement surgery on the inflammatory gene transcriptome of circulating monocytes in older patients. The fact that most of the affected genes were downregulated implies there is a period of immune hypoactivity or paralysis in such patients after surgery. A subset of 21 differentially expressed genes were common across procedures, highlighting these transcripts as a molecular signature of the immune response to surgery in this population and cell type. While a larger data set is required to establish an association of these cellular events with postoperative outcomes, the data identify molecular targets for development as potential risk biomarkers and/or immunotherapeutics to improve postoperative outcomes in this high-risk age group.

## Figures and Tables

**Table 1:** List of 21 DEGs common to surgery independent of the procedure. BH-adj. *p*-val: Benjamini–Hochberg adjusted *p*-values for false discovery rate; log2FC: log2(fold change).

	Hip or Knee Replacement		Spinal Surgery		Combined Analysis	
	BH-adj. *p*-val	log2FC	BH-adj. *p*-val	log2FC	BH-adj. *p*-val	log2FC
FCER1A	2.13 × 10^−12^	−2.35	1.64 × 10^−4^	−1.98	2.54 × 10^−14^	−2.17
CD83	2.83 × 10^−9^	−1.89	6.58 × 10^−5^	−1.27	5.94 × 10^−13^	−1.56
IRF4	1.30 × 10^−17^	−1.87	4.53 × 10^−7^	−1.34	1.08 × 10^−21^	−1.6
IFIT1	8.52 × 10^−10^	−1.86	7.32 × 10^−5^	−1.56	5.67 × 10^−14^	−1.72
S100A12	2.70 × 10^−18^	1.82	9.25 × 10^−8^	1.22	1.49 × 10^−24^	1.55
ELK1	5.44 × 10^−6^	−1.73	2.60 × 10^−2^	−1.01	3.10 × 10^−7^	−1.39
PPARG	2.04 × 10^−14^	1.7	1.46 × 10^−9^	1.85	5.56 × 10^−25^	1.77
SIGLEC1	6.55 × 10^−7^	−1.63	4.72 × 10^−3^	−1.08	3.24 × 10^−9^	−1.38
CXCL10	6.51 × 10^−7^	−1.44	4.46 × 10^−3^	−1.32	2.33 × 10^−8^	−1.37
OAS3	1.53 × 10^−10^	−1.39	2.46 × 10^−4^	−1.14	2.24 × 10^−13^	−1.27
HLA-DPB1	2.74 × 10^−18^	−1.39	1.59 × 10^−6^	−1.2	1.17 × 10^−22^	−1.3
IFIT2	2.13 × 10^−7^	−1.39	6.46 × 10^−4^	−1.2	4.43 × 10^−10^	−1.29
CCL2	1.98 × 10^−5^	−1.36	1.30 × 10^−5^	−1.43	1.48 × 10^−10^	−1.4
SERPING1	6.62 × 10^−7^	−1.34	2.08 × 10^−2^	−1.08	6.94 × 10^−7^	−1.21
HLA-DPA1	6.79 × 10^−17^	−1.29	4.80 × 10^−7^	−1.2	9.90 × 10^−23^	−1.25
IL1R2	2.10 × 10^−5^	1.27	8.50 × 10^−3^	1.6	1.94 × 10^−6^	1.46
HLA-DMB	5.36 × 10^−16^	−1.24	4.63 × 10^−8^	−1.02	1.44 × 10^−23^	−1.14
ISG15	5.60 × 10^−8^	−1.21	1.60 × 10^−4^	−1.01	5.51 × 10^−11^	−1.11
STAT1	2.69 × 10^−13^	−1.2	1.13 × 10^−4^	−1.13	5.57 × 10^−15^	−1.17
HLA-DRA	4.94 × 10^−17^	−1.14	6.12 × 10^−8^	−1.05	1.49 × 10^−24^	−1.1
PTGDR2	1.29 × 10^−10^	−1.08	2.94 × 10^−6^	−1.39	1.75 × 10^−15^	−1.23

## Data Availability

Deidentified participant data that underlie the results reported in this manuscript will be shared beginning 24 months and ending 36 months after article publication pending submission to and approval by the senior authors of a methodologically sound proposal. Data requestors will also be required to sign a data access agreement.

## Supplementary Materials

The following supporting information can be downloaded at: https://www.mdpi.com/article/10.3390/jcm12196271/s1, Table S1: Description of Patient Clinical Characteristics; Table S2: Procedure and anesthesia type.
