# Supplementary material for: Geriatric Surgery Produces a Hypoactive Molecular Phenotype in the Monocyte Immune Gene Transcriptome
Source: J Clin Med. 2023 Sep 28;12(19):6271. doi: 10.3390/jcm12196271 (PMC10573997; doi:10.3390/jcm12196271)
Supplement: Supplementary file 1 [file jcm-12-06271-s001.zip › jcm-2587446-supplementary.pdf]

Supplementary Table 1. Description of Patient Clinical Characteristics

**Characteristic**	**Hip or Knee (n = 19)**	**Spinal (n = 16)**	
Age, years	75.6 +/- 5.4	74.6 +/- 4.5	p = 0.55
Weight, kg	85.3 +/- 22.3	82.7 +/- 23.6	p = 0.74
Body Mass Index	30.8 +/- 7.3	28.2 +/- 5.1	p = 0.23
Female, n (%)	12 (63.2)	6 (37.5)	p = 0.14
Education*, n (%)			
High School Degree	7 (37)	5 (33)	χ^2 = 0.05,
College Degree or higher	12 (63)	10 (67)	p = 0.83
Estimate blood loss, mL	416.3 +/- 405.0	278.8 +/- 328.3	p = 0.31
LOS, days			W = 102,
Median (Q1, Q3)	4 (3, 4.5)	4.5 (4, 6)	p = 0.09
6-month mortality rate	0	0	

LOS, length of stay; χ^2, Chi-square test; *W,* Mann-Whitney (Wilcoxon for rank sum) test.

* one person refused to answer

Supplementary Table 2. Procedure and anesthesia type

	Hip or Knee (n = 19)	Spinal (n = 16)
Procedure types and indices (n)	Knee arthroplasty (10)<br>Hip arthroplasty (6)<br>Anterior hip replacement (3)	Tier 2 (8)<br>Tier 3 (8)
Anesthesia type, n (%)		
General	10 (53)	16 (100)
Regional	6 (32)	
Combined	2 (10)	
Monitored anesthesia care	1 (5)	

Spinal surgery invasiveness classifications:

Tier 2 = lumbar laminectomy, anterior cervical, minimally invasive, foraminotomy, facetectomy

Tier 3 = lumbar fusion, trauma, post cervical.
